# Microbial threats and sustainable solutions for molluscan aquaculture

**DOI:** 10.1093/sumbio/qvae002

**Published:** 2024-01-19

**Authors:** Emily Kunselman, Kara Wiggin, Rachel E Diner, Jack A Gilbert, Sarah M Allard

**Affiliations:** Scripps Institution of Oceanography, University of California, San Diego, La Jolla, CA 92037, United States; Scripps Institution of Oceanography, University of California, San Diego, La Jolla, CA 92037, United States; Department of Biological Sciences, University of Memphis, Memphis, TN 38111, United States; Scripps Institution of Oceanography, University of California, San Diego, La Jolla, CA 92037, United States; Department of Pediatrics, University of California, San Diego, La Jolla, CA 92093, United States; Scripps Institution of Oceanography, University of California, San Diego, La Jolla, CA 92037, United States; Department of Pediatrics, University of California, San Diego, La Jolla, CA 92093, United States

**Keywords:** aquaculture, sustainable, diseases, microbial ecology, biocontrol, mollusc

## Abstract

Aquaculture is responsible for producing almost half of the world’s seafood. As the global climate changes and population continues to increase, we must prepare for increased disease in aquatic animals, a risk compounded by high-density aquafarms that are necessary to keep up with demand. This review will highlight major microbial threats to aquaculture and current and alternative solutions to these threats with consideration for the accessibility of the proposed solutions. Molluscs are ideal for sustainable aquaculture because they require less inputs than most other protein sources, and through filter feeding, they improve local ecosystem health. However, they are also plagued by microbial diseases, and rising water temperatures will only exacerbate this problem by enhancing pathogen survival, range, and growth. At the same time, microbial treatments hold great promise for reducing disease burden and increasing yield and food safety. In order to combat threats to sustainable aquaculture, it is critical to monitor and predict microbial behavior in coastal water and animal populations, explore sustainable microbial treatment options such as probiotics and phage therapy, reduce reliance on antimicrobials, and develop mitigation strategies through partnership with mollusc farmers, government regulators, industry, academic researchers, and indigenous peoples.

**Sustainability Statement**

The solutions reviewed here are proposed to protect and preserve our valuable marine resources and promote the sustainable farming practices of important food supplies and ecological keystone species. Specifically, the sustainable growth of mollusc aquaculture promotes UN Sustainable Development Goals 2 (Zero hunger) and 14 (Life below water) through improving food security while conserving marine resources. Molluscs are a benefit to both their environment and their consumers, providing ecosystem services such as water filtration and nutritious meals full of vitamins and minerals. The goal of this review is to summarize the role of microorganisms in sustainable mollusc aquaculture and to encourage the development and use of beneficial microbial treatments to prevent animal and human disease.

## The economic importance and sustainability of molluscan aquaculture

Farmed molluscs are an increasingly important component of our global food supply. In 2020, about half of the global aquatic animal production came from aquaculture, with the other half coming from capture fisheries (FAO 2022). In 2022, 17.7 million tonnes of molluscs were produced in aquaculture globally, totaling ∼29.8 billion USD (FAO 2022). Noncephalopod molluscs, and specifically marine bivalves, including scallops, clams, oysters, mussels, and abalone, constitute the majority of molluscan aquaculture. In some countries, marine bivalves are farmed more than any other marine species (FAO 2022). For example, 86.9% of aquaculture in New Zealand, 75.4% in France, and 74.8% in Spain are from marine bivalves (FAO 2022).

Not only are noncephalopod molluscs an integral part of the global economy, but they are also one of the most sustainable food options on the market (Willer et al. 2021). Most molluscs, notably the bivalves, are filter feeders; thus, they do not require added food such as finfish aquaculture and land-based agriculture, significantly reducing resources required for production. Because of their feeding behavior, molluscs provide essential ecosystem services, such as the removal of nitrogen and phosphorus (Schatte Olivier et al. 2020), and anthropogenic pollutants such as heavy metals (Bonnard et al. 2020). Leftover shells can be used for agriculture or construction applications (Bonnard et al. 2020) and are culturally valuable for jewelry and decoration. Considering the growing demand for sustainable food sources (Willer et al. 2021), it is important to review the state of molluscan aquaculture and determine the strengths and weaknesses of current farming practices as well as threats and opportunities for the industry. Molluscan aquaculture is a promising opportunity to work toward Zero hunger while preserving Life below water, which are two of the UN Sustainable Development Goals.

In this review, we examine molluscan aquaculture through the microbial lens to identify the risks posed by microbes and the solutions they may offer to bolster molluscan production and disease resilience. We further discuss the accessibility of microbial interventions in molluscan aquaculture and propose methods for increasing the range of communities that may benefit from these applications.

## Microbial threats to sustainable molluscan aquaculture

While harnessing beneficial microbes (bacteria, viruses, eukaryotes) may hold substantial promise in sustainable molluscan aquaculture, some microorganisms pose serious risks to both production and human health. Molluscs can be infected by a suite of bacterial, viral, and protist pathogens (Table 1) that lead to mass mortality events and can accumulate in mollusc tissue, creating risks for human consumption. These diseases spread faster when animals are stocked at the higher densities required for aquaculture (Bruto et al. 2017). In 2014, it was estimated that microbial threats cost the aquaculture industry $6 billion (World Bank 2014), and in 2018, economic losses (e.g. lost wages and medical care) in the USA due to foodborne illness reached over $17 billion, with over $3 billion from shellfish-derived pathogens alone [US Department of Agriculture (USDA), Economic Research Service (ERS) 2023]. Unless these microbial threats can be managed, these costs will continue to accrue because increased disease prevalence leads to reduced product and farm closures.

**Table 1.** Commonly cultured mollusc species and diseases that affect them.

Species	Animal pathogens	Human pathogens
**Oysters**		
*Crassostrea gigas* (Pacific oyster) *C. virginica* (eastern oyster) *Ostrea edulis* (flat oyster) *Spondylus* spp. (spiny oyster) *Pinctada* spp. (pearl oyster)	Ostreid Herpes Virus (OsHV-1)* Perkinsus marinus (Dermo)* Perkinsus olseni* Vibrio spp. *Roseovarius crassostreae* (Roseovarius oyster disease/juvenile oyster disease) *Haplosporidium nelsoni* (MSX) *Marteilia refringens* Bonamia*(Gill necrosis virus) *Nocardia crassostreae* (Pacific oyster nocardiosis)	*Vibrio* spp. Norovirus Hepatitis A
**Mussels**		
*Mytilus edulis* (blue) *M. trossulus* (Bay) *M. galloprovincialis* (Mediterranean) *Perna viridis* (green) *M. chilensis* (chilean) *P. canaliculus* (green-lipped) *M. coruscus* (hard-shell)	*M. refringens* *Vibrio* spp.	Norovirus Hepatitis A
**Clams**		
*Mercenaria mercenaria* (northern quahog) *Venerupis philippinarum* (Manila) Cardiidae (cockles) Veneridae (venus) *Siliqua patula* (razor) *Ruditapes decussatus* (carpet shells) *Mya arenaria* (soft shell) *Protothaca staminea* (little neck) *Tridacna gigas* (giant) *Panopea generosa* (geoduck)	Quahog Parasite Unknown (QPX) *Marteilia refringens* *Perkinsus olseni* *V. tapetis* (brown ring disease)	Norovirus Hepatitis A
**Scallops**		
*Placopecten magellanicus* (American) *Pecten maximus* (Great Atlantic/king) *Aequipecten opercularis* (Queen) *Argopecten irradians* (Bay) *Chlamys farreri* (Chinese) *Patinopecten yessoensis* (Yesso)	*Francisella halioticida* *Marteilia* spp. *V. splendidus* *V. natriegens*	
**Abalone/Snails**		
*Haliotis rufescens* (red abalone) *H. tuberculata* (green ormer) *H. midae* (South African/perlemoen)	Herpesvirus (ganglioneuritis)* Abalone Withering Syndrome (CaXc)* Vibrio spp.	

* Indicates that the World Organization for Animal Health lists this disease on their website.

** Diseases are listed in no particular order. Disease listed does not correspond to the mollusc in its same row.

As filter feeders and grazers, molluscs are continually exposed to new microbes and potential pathogens from their environment. Molluscan-associated microbiomes are often host and tissue specific, offering immune protection to their host through competition and aiding in food digestion (Pierce and Evan Ward 2018). However, the composition of microbial species that colonize the mollusc tissues and shell is also influenced by water temperature, oxygen exposure, water depth, food availability, and even certain microbial pathogens that can destabilize the microbial ecology of these host-associated environments (Khan et al. 2018, Li et al. 2018, 2019, Simons et al. 2018, Offret et al. 2020). If a mollusc is stressed by any of these factors, the microbiome is also likely to shift into an altered state, sometimes referred to as dysbiotic, which simply means that the microbiome and host are no longer in a beneficial symbiosis. For example, when oysters are co-exposed to both OsHV-1 and *Vibrio* pathogens, the OsHV-1 virus weakens the host immune system, allowing the *Vibrio* pathogen to take hold, ultimately leading to mortality (de Lorgeril et al. 2018). Infection with the bacterial pathogen Candidatus *Xenohaliotis californiensis* in abalone disrupts digestive gland morphology, causing the abalone to starve and shrivel, which is why the disease is termed “Withering Syndrome” (Crosson et al. 2014). Many *Vibrio* species are generally harmless members of the oyster microbiome, but may become pathogenic upon acquisition of virulence genes, such as the case for *V. crassostreae* (Bruto et al. 2017). High stocking density on farms has been linked to increased disease intensity, and horizontal gene transfer may also contribute to this as it offers a plethora of opportunities for microbes to transfer back and forth between hosts (Bruto et al. 2017, Fox et al. 2020).

In addition to the risks microbes pose to molluscs, they also pose a risk to their human consumers. Historically, shellfish are responsible for the majority of seafood-related disease outbreaks in the USA (Oliveira et al. 2011), primarily derived from viruses and bacteria that accumulate in shellfish tissue. *Vibrio* species, including *V. vulnificus* and *V. parahaemolyticus*, are the leading cause of seafood-related bacterial infections globally, also known as vibriosis, resulting in gastroenteritis, vomiting, diarrhea, and, in some cases, death. Incidence of noncholera *Vibrio* species has increased in the USA since 2006 unlike all other major foodborne illnesses, with *V. parahaemolyticus* responsible for the majority of these cases (Baker-Austin et al. 2018). *Vibrio parahaemolyticus* infection rates are likely increasing due to climate change. As this species thrives in warmer temperatures, particularly >15°C, global oceanic temperature rise is encouraging rapid range expansion northward (Baker-Austin et al. 2017, Abanto et al. 2020). In the USA, *V. vulnificus* infections are less common than *V. parahaemolyticus* infections but more serious, with >80% of cases resulting in hospitalization and >30% of cases resulting in death (Newton et al. 2012). *Vibrio vulnificus* is the most fatal foodborne pathogen in the USA, accounting for the majority of seafood-related deaths (Campbell et al. 2022). Unlike most bacterial outbreaks, viral outbreaks result from exclusively anthropogenic-derived viruses. The most common implicated species are norovirus [exclusively genogroups I and II; most common genotypes include GI.4, GII.4, GII.b, and GII.3 (Razafimahefa et al. 2020)] and hepatitis A (Bellou et al. 2013), which accumulate in shellfish through wastewater discharge, sewage overflow, and stormwater discharge during heavy rainfall, or through handling by shellfish harvesters and processors (Bellou et al. 2013, Gyawali and Hewitt 2020), without adverse side effects on the health of the shellfish host. Most outbreaks occur from oysters, often served raw, or clams, often served undercooked, failing to eliminate viral threats to human consumers (Bellou et al. 2013). Even cooking or pickling contaminated shellfish will not always be enough to completely destroy human pathogens.

Rising water temperatures have been linked to increased spread and virulence of several microbial pathogens of molluscs (Colleen and Burge 2020), most notably the *Vibrio* species mentioned above. High temperatures allow for enhanced pathogen growth, especially within the *Vibrio* genus (Tantillo et al. 2004). High temperatures also cause heat stress for the host, resulting in physiological shifts such as immunosuppression (Dang et al. 2012, Rahman et al. 2019), which can make the host more susceptible to infection. Under high-temperature conditions, abalone are more at risk of abalone withering syndrome (Moore et al. 2000) and *V. harveyi* infections (Travers et al. 2009). Likely, these tight links to temperature predict an even higher disease burden in molluscan aquaculture under future climate-change scenarios. Furthermore, associated challenges of climate change include ocean acidification, which can lead to reduced growth and reproduction (Barton et al. 2012, Burge et al. 2014). The burden of these diseases must be taken into account when farmers are planning their growing seasons, as shellfish are at the mercy of the natural seawater conditions and have limited protection from potentially pathogenic microbes in the water column.

## Current solutions to mitigate microbial threats in molluscan aquaculture

The threats of economic loss and food contamination due to microbial pathogens are long-standing obstacles in all food production systems. Many solutions have been presented and are practiced frequently in molluscan aquaculture to eliminate or at least minimize the amount of harmful microbes at every step of the production chain. We review here some options and their associated benefits and pitfalls.

At the hatchery stage of molluscan aquaculture, pathogen control consists primarily of mechanical filters that prevent the entry of pathogens into the farm. Filtration with sand filters or pore size filters down to 0.22 μm, ultraviolet light sterilization, and ozonation are all used to reduce or remove external contaminants from incoming water supplies, so as to prevent potential pathogens from entering a mollusc production facility. More recently, the practice of ultrafiltration has become popular as it removes bacteria, particularly *Vibrio* species, more efficiently than other filtration methods and UV disinfection (Cordier et al. 2021). The ability to maintain healthy hatchery systems depends greatly on the financial and technological availability of the farmer, as the more advanced mechanical controls can be a financial burden.

Once the molluscs are successfully settled from their larval stage and start to grow their shell, they are typically moved into the natural environment and must provide their own innate defense against pathogens. There are limited options available to farmers to prevent molluscan disease and mortality once they are at the whim of the environment, and diseases are entirely dependent on the location and physicochemical parameters of the water (Burge et al. 2014). Because molluscs lack an adaptive immune system, they are not a good candidate for vaccine development; thus, approaches such as antibiotic therapy are one of the only viable options for controlling the spread of disease.

Data on the use of antibiotics in molluscan aquaculture are very limited because they are not often reported and there is a lack of regulation on antibiotic usage in some countries (Schar et al. 2020, Hossain et al. 2022). Control of abalone withering syndrome, a disease of concern in captively bred abalone, is a common case of antibiotic use in a strictly closed aquaculture setting using oxytetracycline baths (Friedman et al. 2007). This disease has not been detected in wild abalone; thus, antibiotic use is strictly applied in closed laboratory settings to prevent the spread of this pathogen from outplanted individuals.

**Figure 1.**
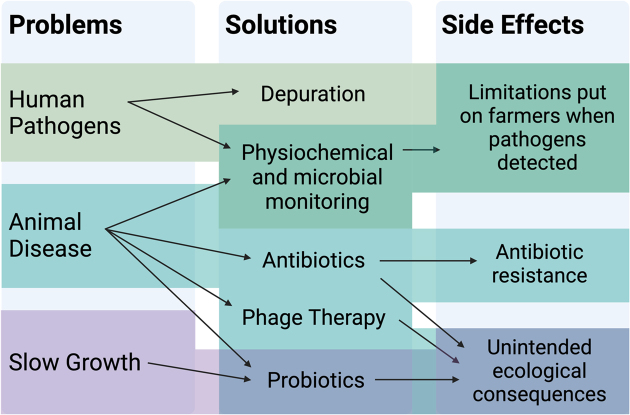
Weighing the options. Current and emerging solutions to problems encountered by aquaculture and potential negative side effects of those solutions. Created with BioRender.com

Antibiotic use in molluscan aquaculture has significant drawbacks, as demonstrated frequently in finfish aquaculture. Aquaculture conditions encourage the transfer of resistance genes between both animals and microbes, and antibiotic-resistant strains can persist in the environment indefinitely (Watts et al. 2017, Hossain et al. 2022) (Fig. 1). Many examples of antibiotic use in aquaculture have been documented, e.g. in the Bohai Sea in China (Li et al. 2012) and in a clam hatchery in Spain that led to antibiotic-resistant *Vibrio* (Dubert et al. 2016). Antibiotic resistance is a growing and important threat to human health globally (Murray et al. 2022). Antibiotic resistance was documented in a scallop hatchery, where *Pseudomonas* and *Pseudoalteromonas* strains from larval scallops were found to be resistant to florfenicol, chloramphenicol, streptomycin, and co-trimoxazol (Miranda et al. 2013). This case is an early warning against the misuse of antibiotics in hatchery systems as a disease mitigation plan.

At the harvesting and processing stages, the primary concern is controlling human pathogens. Many operations employ preharvest and immediate postharvest control measures such as immediate cooling to prevent potential pathogen growth. Some operations utilize methods to further minimize pathogen load, such as depuration, prior to sale. Depuration is the process of purging microbial pathogens through exposure to treated seawater postharvest (Fig. 1). This method can be time-, cost-, and resource-intensive, thus limiting the accessibility for smaller aquaculture farms. Depuration can be highly effective against enteric bacteria (Love et al. 2010), but physicochemical parameters, such as salinity, may dictate the effectiveness against other bacteria. For example, *V. vulnificus* and *V. parahaemolyticus*, human pathogens found in oysters, are only efficiently removed under high salinities and low temperatures after at least 3 days because these conditions are detrimental to the pathogen’s growth (Lewis et al. 2010, Campbell et al. 2022). Viruses are especially difficult to depurate from shellfish, typically taking much longer to remove than enteric bacteria (Love et al. 2010). Testing protocols are available for these pathogens, such as the ISO 15216-1 quantitative real-time reverse transcription polymerase chain reaction (RT-PCR) method for norovirus, and show a significant reduction following depuration but not complete removal (Rupnik et al. 2021). Maintaining these parameters and meeting strict depuration guidelines can be very challenging and costly, and may also result in mollusc mortalities when conditions are too stressful for molluscs themselves; thus; it is not practiced as strictly in the USA as it is in other countries (Campbell et al. 2022). Although depuration is an important component of disease mitigation, i.e. highly effective for certain pathogens, other strategies are necessary for comprehensive disease management. Due to the disparate effectiveness across organisms and logistic and financial challenges of depuration, there is an urgent need for other accessible and effective approaches to molluscan disease management.

## How microbes could be used to improve sustainable molluscan aquaculture

Microbes are often viewed negatively in aquaculture as their ability to cause sickness in both the animal and human consumers is a major risk and therefore nonbeneficial for business. However, microbes have the potential to benefit aquaculture through mechanisms such as biocontrol of pathogens and may even increase animal health and growth via probiotics. Therefore, this section is focused on supporting the use of microbial therapy and treatments in sustainable mollusc aquaculture.

Probiotics are live microorganisms, which when consumed live in sufficient quantities produce a measurable health benefit. While predominantly considered a human therapy, they are now being more widely adopted in animal husbandry (Hossain et al. 2017, Ringø 2020). Probiotics are supplemented into an animal’s diet to enhance their immune system, strengthen gut microbiomes, and increase antimicrobial mechanisms (Hossain et al. 2017, Ringø 2020). Probiotics compete with potential pathogens through either direct or indirect competition for resources. They are also able to colonize the host; e.g. probiotic strains can integrate into the scallop larval microbiome and offer lasting protection against multiple pathogens (Riquelme et al. 2000). There are already numerous examples of probiotics that are successfully used in mollusc hatcheries as a primary treatment against pathogen accumulation. Probiotics using strains of *Aeromonas, Phaeobacter, Bacillus, Alteromonas*, and *Pseudoalteromonas* species have been applied to oyster hatcheries to increase resistance and protection against *Vibrio* pathogens such as *V. splendidus, V. coralliilyticus, V. tubiashii, V. harveyi*, and *V. pectenicida* (Kesarcodi-Watson et al. 2012a, b, Sohn et al. 2016, Grandiosa et al. 2018, Stevick et al. 2019, Ringø 2020, Madison et al. 2022). However, probiotics differ in their effectiveness between different species of shellfish. For example, probiotic treatment with *Phaeobacter inhibens* and *B. pumilus* did not confer any advantage against *Vibrio* accumulation in northern quahogs, razor clams, and blue mussels, while oysters and bay scallops showed increased protection when administered the same probiotics (Sohn et al. 2016). While potentially valuable, probiotic administration can be detrimental if applied incorrectly. For example, high-dose multistrain probiotic treatment with *Alteromonas macleodii* and *Neptunomonas* sp. significantly decreased larval survival in the greenshell mussel (Kesarcodi-Watson et al. 2012a). Multiple tests are required per species to determine the most effective method of probiotic exposure that works synergistically with the host microbiome to confer protection against disease (Kesarcodi-Watson et al. 2012a).

Aside from disease protection, some probiotics also promote faster growth in molluscan culture facilities. Multiple probiotics such as *Exiguobacterium* JHEb1, *V*. JH1, *Enterococcus* JHLDc, *B. amyloliquefaciens, Enterobacter ludwigii, Pediococcus acidilactici*, and *V. midae* used on abalone have increased their growth rate, which is valuable for the sustainable production of a species that typically takes 4 years to reach market size (Grandiosa et al. 2018, Amin et al. 2020, Moonsamy et al. 2020). Most probiotics have been investigated for use in the hatchery setting, where inflow and outflow water can be manipulated. The use of probiotics needs to be carefully considered if they are implemented in natural environments, such as grow-out tidal zones, because they could also impact local microbial ecology. Still, probiotics are a promising alternative to antibiotics and are still greatly underutilized in mollusc aquaculture to increase disease resistance and support faster growth (Fig. 1). These early success suggest that the advantages likely outweigh the cost of developing and implementing probiotic therapies in an aquaculture.

**Figure 2.**
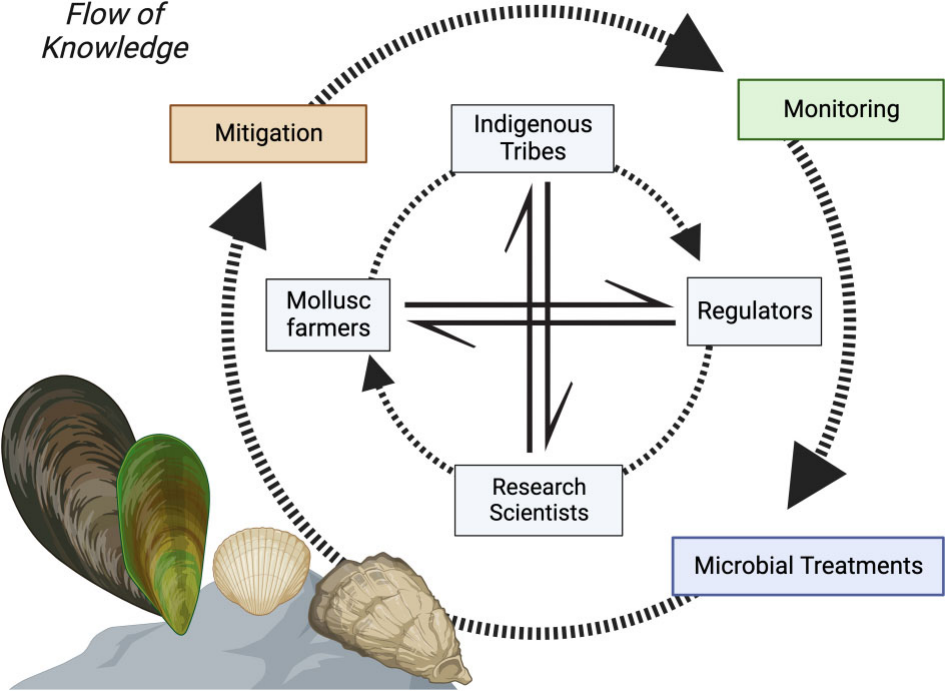
Modern sustainable solutions for molluscan aquaculture. How knowledge and information about effective strategies for protecting mollusc aquaculture should flow between contributors (inner ring), and targets for microbially mediated solutions for disease management (outer ring). Created with BioRender.com

Phage therapy is another microbially mediated intervention that has seen some promising results in mollusc aquaculture. Naturally isolated phages have been found to reduce the effects of rickettsial disease withering syndrome on black abalone (Friedman et al. 2014), controlling the spread of the disease in the wild. Phages have also been found to be effective against human pathogens such as *V. parahaemolyticus* (Jun et al. 2014, Chang et al. 2016). However, studies have shown that phages have varying levels of broad-spectrum defense, may lead to resistance in target bacteria, and may be sensitive to some physicochemical factors such as temperature. Furthermore, more research is needed into the safety and public perceptions of adding phages to food (reviewed in Pereira et al. 2021). From a sustainability perspective, phage therapy has many advantages over other control methods such as antibiotic use and depuration practices (Fig. 1). Unlike antibiotic use, phages have almost no effects on the health of the host and allow the natural microbiome to flourish (Pereira et al. 2021). To support the growth of sustainable molluscan aquaculture, increasing development and use of both probiotic and phage therapies will require effort on the part of every vested party in aquaculture microbial management—(i) researchers, to continually develop the therapies targeting pathogens of concern; (ii) regulators, to approve the use of these techniques for use and to monitor their effectiveness; and (iii) mollusc farmers, to implement these strategies and use them to increase the sustainability of their farms (Fig. 2).

In addition to harnessing microbes for growth and protection, concurrent monitoring and modeling of pathogenic microbes in farming areas is essential to predict and plan for potential disease outbreaks. Monitoring must be continuous to capture seasonal and annual dynamics for future modeling efforts (Nenciu et al. 2020); however, monitoring methods often require significant resources in both time and money. Current monitoring techniques mainly rely on culture-based methods for bacteria, which are more cost-effective, but fail to detect viruses and unculturable bacteria, which require expensive, though more reliable, molecular methods such as quantitative PCR (qPCR) (Muniain-Mujika et al. 2003) and droplet digital PCR (ddPCR) (Persson et al. 2018). While resource expenditure may be high to collect and test samples for pathogens, continuous monitoring provides farmers with critical information they need to plan their growing and harvesting seasons to maintain a healthy stock and avoid disease outbreaks among their consumers. Additionally, if and when a pathogen is detected, there should be a collaboration between researchers, farmers, and government regulators to find solutions to these microbial risks and identify safe thresholds (see Fig. 2).

Environmental monitoring of biotic and abiotic factors is an important supplement to pathogen monitoring. In more resource-limited settings, it can also be a cost-effective, albeit less reliable, method of pathogen risk assessment. For example, temperature and precipitation (and thus salinity) patterns can be used to predict fecal coliforms, *Vibrio* spp., and viral contamination in molluscs (Elston et al. 2008, Jeamsripong et al. 2018, Green et al. 2019). *Vibrio* spp. may also be tightly coupled to phytoplankton composition (Lopez-Joven et al. 2018). Co-monitoring for harmful algal species and pathogens can be conducted to maximize sampling efforts. Lower-cost monitoring can include the detection of indicator species such as *Escherichia coli*, which have reliable, cheap, and fast methods of detection, and can signal the deterioration of the surrounding water and animal health (Bougeard et al. 2011). In addition, biotic factors such as oyster age and size can influence susceptibility to pathogens as in the case of certain *Vibrio* spp. and OsHV1, which are more severe in younger life stages (King et al. 2019). Modified field practices can also influence disease susceptibility, such as altered growing heights or periodic transplanting of stock (King et al. 2019). Co-monitoring of multiple threats, both abiotic and biotic, and utilizing the wealth of research on best growing practices to avoid these threats, shows promise for effective microbial risk assessment and management.

## Sustainability extends beyond the environment: inclusion and access for microbial tools in aquaculture

The sustainable solutions proposed in this review center around using microbes to advance aquaculture production and prevent disease outbreaks. However, the research time and costs associated with launching programs to test probiotics and establish long-term monitoring are often not tangible for mollusc farmers alone. The development of innovative microbe-centered solutions requires support from funding agencies that award research grants to scientists to carry out the necessary experiments. It is crucial for these scientists to include other vested parties in the design, execution, and dissemination of research to advance sustainable molluscan aquaculture, to ensure that these sustainable solutions are accessible, reasonable to implement, and effective (Fig. 2).

The immediately obvious step is to include farmers in the grant process, provide money for any time or resources that they provide, and ensure that they are the direct beneficiaries of the results. In addition, it is important to consider the past and present stewards of the area and ensure indigenous communities are part of the research and decision-making process (Fig. 2). Prior to colonization, indigenous communities sustainably harvested shellfish for millennia, and shellfish remain a culturally and economically significant resource for many indigenous communities around the globe (Reeder-Myers at al. 2022). Indigenous perspectives are valuable for developing the sustainable use of marine resources, as has been demonstrated in New Zealand (Harmsworth and Awatere 2013) and British Columbia (Joyce et al. 2010), where indigenous expertise related to land-use and ecosystem assessments has enhanced ecosystem sustainability. Second, indigenous communities should receive first rights to sustainable aquaculture permits in their historical land (Joyce et al. 2010), and be on the receiving end of any research done by scientists on the advancement of sustainable molluscan aquaculture (Fig. 2).

As a best practice in aquaculture research, data should be treated as shared knowledge for all members of society that may use the aquacultured resource. Knowledge should be deposited in public databases, but also converted into digestible pieces of scientific communication to benefit the broader community.

## Conclusion

Molluscan aquaculture is critical to our future food systems, and there is great opportunity for advancement in aquaculture technology that encourages the sustainable expansion of this important food resource. Despite its sustainability advantages, the industry faces challenges from microbial disease, exacerbated by climate change. This review advocates for a multipronged approach to address these challenges. Probiotics and phage therapy (as alternatives to antibiotics) show promise in mitigating disease impact and have an added benefit of enhancing yields. Continuous monitoring of microbial behavior in coastal areas is necessary, requiring collaboration among farmers, regulators, researchers, and indigenous communities. Finally, inclusivity is essential for the sustainability of molluscan aquaculture. We promote open data sharing, allocating funding from research grants to farmers who participate in scientific research, and equal decision-making and opportunity for indigenous communities.

## Data Availability

There are no new data associated with this article.
